# Two Effective Routes for Removing Lineage Restriction Roadblocks: From Somatic Cells to Hepatocytes

**DOI:** 10.3390/ijms160920873

**Published:** 2015-09-01

**Authors:** Chenxia Hu, Lanjuan Li

**Affiliations:** Collaborative Innovation Center for Diagnosis and Treatment of Infectious Diseases, State Key Laboratory for Diagnosis and Treatment of Infectious Diseases, School of Medicine, First Affiliated Hospital, Zhejiang University, Hangzhou 310058, China; E-Mail: 11318093@zju.edu.cn

**Keywords:** induced pluripotent stem cells, differentiation, reprogramming, hepatocyte, *in vitro*, *in vivo*

## Abstract

The conversion of somatic cells to hepatocytes has fundamentally re-shaped traditional concepts regarding the limited resources for hepatocyte therapy. With the various induced pluripotent stem cell (iPSC) generation routes, most somatic cells can be effectively directed to functional stem cells, and this strategy will supply enough pluripotent material to generate promising functional hepatocytes. However, the major challenges and potential applications of reprogrammed hepatocytes remain under investigation. In this review, we provide a summary of two effective routes including direct reprogramming and indirect reprogramming from somatic cells to hepatocytes and the general potential applications of the resulting hepatocytes. Through these approaches, we are striving toward the goal of achieving a robust, mature source of clinically relevant lineages.

## 1. Introduction

As the largest organ in the human body, the liver performs multiple metabolic functions, including detoxification, glucose metabolism, plasma protein synthesis, and bile production [1]. These metabolic functions are supported by sinusoidal endothelial cells, hepatic stellate cells, Kupffer cells, and cholangiocytes. These cells cooperate to regulate hepatocyte function and maintain homeostasis in humans or animals. However, because of the various causes of hepatic injury, the number of people living with end-stage liver disease is increasing rapidly, and more than one million people die each year from acute and chronic liver diseases across the globe [2]. The need for liver transplantation has increased rapidly, accompanied by an ongoing shortage of donor livers. To reduce the demand for tissue, hepatocyte transplantation has been substituted for whole organ transplantation. However, this approach has not been widely adopted due to a variety of technical reasons, including the inability to monitor graft health and frequent rejection signs [3]. Hepatocytes proliferate poorly *in vitro*, although microenvironments that promote their multiplication are being intensively but unsuccessfully investigated.

**Figure 1:**
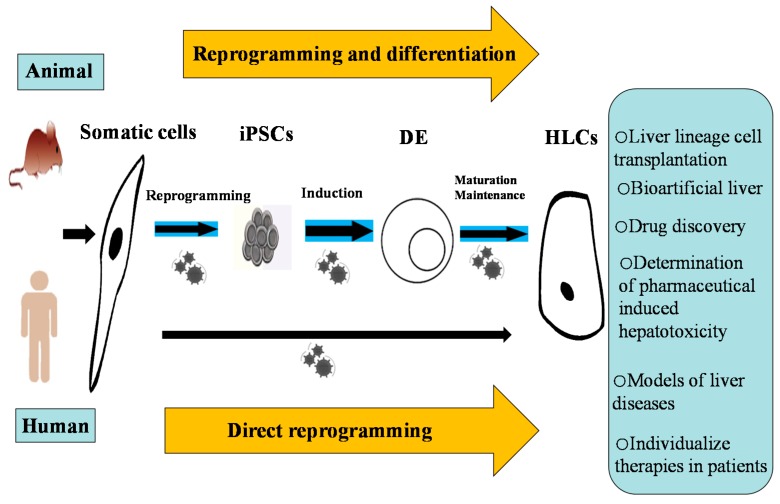
Promoting a unified field in induced pluripotent stem cell (iPSC)-derived HLCs and achieving a robust, mature source of clinically relevant lineages.

Pluripotent stem cells, which can self-renew, maintain genetic stability, and possess multilineage potential *in vitro*, have attracted attention worldwide. Cell lines derived from hepatocellular carcinoma or generated through SV40 large T antigen transformation have enabled the expansion and creation of *in vitro* model systems [4], but their malignant backgrounds and requirements for non-physiological manipulations have inhibited their clinical usage. Embryonic stem cells (ESCs) derived from the inner cell mass of mammalian blastocysts have been deemed as ideal candidates for regenerative medicine but have resulted in ethical concerns and incompatibility with the immune system. Adult tissue-derived stem cells, which are plentiful without using embryonic materials, can be easily extracted but possess innate limitations in stem cell potency and therapeutic potential. Induced pluripotent stem cells (iPSCs) were first generated by Yamanaka and colleagues following the forced expression of four transcription factors (*OCT3/4*, *SOX2*, *C-MYC*, and *KLF4*) in somatic cells [5]. Upon manipulation of their culture conditions *in vitro* or their transplantation into mice, iPSCs can be differentiated into numerous endodermal lineages, including hepatocytes [6]. iPSC-derived hepatocyte-like cells (HLCs) can be utilized in disease modeling, drug toxicity testing, and autologous cell therapies that would avoid immune rejection and enable the correction of genetic defects. In this review, we provide a summary of two effective routes including direct reprogramming and indirect reprogramming from somatic cells to hepatocytes and the general potential applications of the resulting hepatocytes. Through these approaches, we are advancing toward the goal of achieving a robust, mature source of clinically relevant lineages (Figure 1).

## 2. Reprogramming Somatic Cells to Induced Pluripotent Stem Cell (iPSCs)

Most studies have focused on generating iPSCs from somatic cells and have developed multiple routes to improve the efficiency of this process in different cell types. To reprogram efficiently and safely, several aspects must be considered. First, the reprogramming efficacy varies according to the cell type; thus, the choice of cell type may initially determine the transition efficiency; Second, reprogramming systems such as viral vectors, non-viral vectors, direct protein transduction and other new systems exhibit different efficiencies; Third, an optimized combination of reprogramming factors can enhance the reprogramming efficiency; Fourth, when culturing *in vitro*, the environment plays a crucial role in maintaining the function and potential usage of iPSCs; Last, genetic manipulation may result in mutations and abnormal karyotypes. By retroviral [7] or lentiviral [8] transduction of transcription factors, iPSCs were derived from somatic cells; however, this approach was associated with genome insertion and tumorigenicity risk. Human iPSC lines were later established from fibroblasts using adenoviral vectors, but extremely high viral titers had to be used in this reprogramming system [9]. Sendai virus, an RNA virus that does not integrate into the genome, is also capable of delivering reprogramming factors [10]; however, this system is difficult to manipulate and requires more than 15 passages to eliminate viral transgene expression. Non-integrating vectors including plasmids [11], episomal vectors [12], and minicircles [13] will not result in chromosomal abnormalities, but the efficiency is extremely low. An mRNA-based integration-free technique has been successfully used to generate iPSCs from adipose tissues and established personalized regenerative medicine within the clinic [14]. Moreover, iPSCs have been established by the direct delivery of recombinant reprogramming proteins [15] and small molecules [16]. Notably, a cocktail of SB431542, PD0325901, and thiazovivin has improved the reprogramming efficiency up to 200-fold [17]. Due to low transduction efficiency and unstable expression, the reprogramming efficiency of transgene-free methods is lower than with viral vectors. Therefore, developing efficient technologies that require less time is an urgent task.

Mouse and human cells can be reprogrammed by the ectopic expression of the transcription factors *OCT3/4*, *SOX2*, *KLF4*, and *C-MYC* [5]. *OCT4* can be replaced by *GATA3*, *GATA4*, and *GATA6*, and *SOX2* can be replaced by *SOX1* and *SOX3* [18]. *BMI1* functionally replaced the three transcription factors *SOX2*, *KLF4*, and *C-MYC* and, along with *OCT4*, generated authentic iPSCs [19]. Samavarchi *et al.* [20] suggested that *NANOG* alone is sufficient to mediate the transition from pre-iPSCs to stably reprogrammed cells. Another study demonstrated that *OCT4* is dispensable in the generation of porcine iPSCs [21]. Furthermore, over-expression or deletion of some transcription factors can affect reprogramming efficacy in addition to the classical transcription factors. For example, over-expression of *MOF* improves the reprogramming efficiency and facilitates iPSC formation [22]. *PRMT5* over-expression in combination with *OCT3/4*, *SOX2*, *KLF4*, and *C-MYC* significantly increased the number of alkaline phosphatase-positive goat iPSCs compared to the four transcription factors alone [23]. *SNAI1* and *SNAI2* play opposite roles in *NANOG*-dependent reprogramming. Ectopic expression of *SNAI1* or depletion of *SNAI2* greatly facilitates *NANOG*-driven reprogramming [24]. Successful reprogramming may depend on a stoichiometric balance of a combination of potential transcription factors.

ESC culture conditions for iPSCs may raise issues of microbial or viral transmission and immunogenicity. Thus, feeder-free culture systems to eliminate contamination are obligatory for scientists to develop clinical-grade iPSCs [25,26,27]. In addition to the culture system conditions, hypoxia will accelerate the reprogramming process [28]; therefore, the optimal O_2_ concentration should be determined [29]. In addition, a three-dimensional (3D) culture system provides a simple, efficient, and time-saving method for the provision of iPSCs at midi-scale [30]. In summary, combining all the optimized conditions such as the suitable cell source; excellent combination of reprogramming factors, non-integrated vectors or excisable vectors; and feeder-free culture systems or 3D culture systems, iPSCs can be safely generated and differentiated to other lineages for research or clinical applications.

## 3. The Hepatic Differentiation Abilities of Different Sources Vary

Many animal models (e.g., mouse [31] and porcine [32]) have been used to investigate the detailed mechanisms of iPSC hepatic differentiation. The reprogramming or differentiation effects of the tissue sources vary. For example, fetal hepatocytes can be reprogrammed much more efficiently than adult hepatocytes [33], and the hepatic differentiation efficiency of peripheral blood cell-derived iPSCs is better than that of adult dermal fibroblast-derived iPSCs [34]. When comparing peripheral blood- and dermal fibroblast-derived iPSCs from the same individuals, variations in hepatic differentiation were largely attributable to donor differences rather than to the original cell types [34]. Similar to other somatic cell lineages, hepatocyte-derived iPSCs were indistinguishable from ESCs with respect to colony morphology, growth properties, pluripotency-associated gene expression, surface marker expression, differentiation potential in embryoid body formation, and teratoma assays. In addition, the resulting iPSCs can directly differentiate into definitive endoderm, hepatic progenitors, and mature hepatocytes [35]. Hepatoblast-derived iPSCs were more efficient in directed differentiation toward the hepatic lineage than adult hepatocyte-derived iPSCs, mouse embryonic fibroblast-derived iPSCs, or mouse ESCs. These cells retained a transcriptional memory (seven up-regulated and 17 down-regulated genes) typical of the original cells; however, most of these differences, including a superior capacity for hepatic redifferentiation, were erased after continuous passaging [36]. Thus, the selection of somatic cell sources may result in different reprogramming efficiencies or differentiation efficiencies according to the various applications.

## 4. The Characteristics of iPSC-Derived Hepatocyte-Like Cells (HLCs)

**Figure 2:**
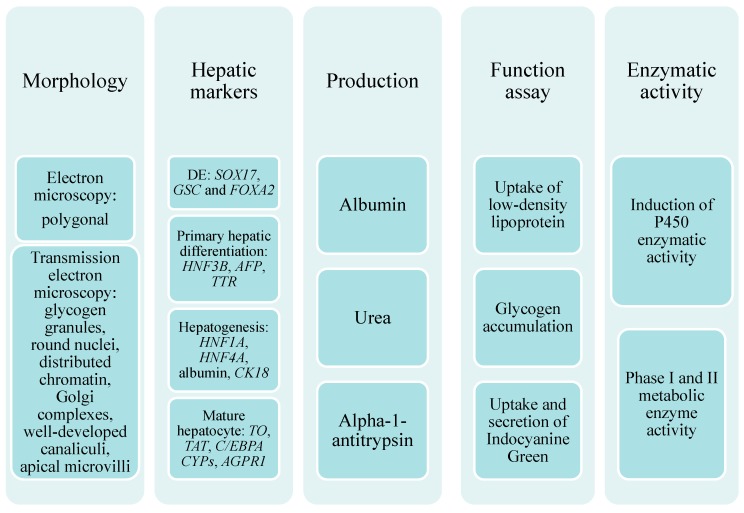
A set of criteria must be met before characterizing iPSC-derived cells as “hepatocyte-like”.

iPSC-derived HLCs present an excellent alternative for addressing availability issues and provide key features of their native counterparts. Indeed, a set of criteria must be met before characterizing iPSC-derived cells as “hepatocyte-like” (Figure 2). Under electron microscopy, the appearance of polygonal cells containing multiple nuclei is suggestive of hepatocytes. Under transmission electron microscopy, glycogen granules within the cells, round nuclei with evenly distributed chromatin and Golgi complexes, well-developed bile canaliculi with apical microvilli, and tight junctions are characteristics of mature hepatocytes [37]. Each phase of differentiation is delineated by specific markers. At the molecular level, the intermediate phase of hepatogenesis is characterized by the expression of *HNF1A*, *HNF4A*, *ALB*, and *CK18*. *HNF3B*, *AFP*, and *TTR* are markers of primary hepatic differentiation, and *SOX17*, *GSC*, and *FOXA2* are well-known markers of definitive endoderm. Finally, mature hepatocytes are defined by the expression of *TO*, *TAT*, *C/EBPA*, *CYPs*, and *AGPR1* [38]. At the protein level, the production of albumin, urea, and alpha-1-antitrypsin and the induction of *P450* enzymatic activity following treatment with specific inducers and substrates to confirm phase I and II metabolic enzyme activity and their functional abilities are commonly tested in each phase of differentiation [39]. The stable expression and function of *CYPs* and transporters in iPSC-derived HLCs for at least one week allows long-term and extensive studies to be reproducibly performed [40]. These cells maintain the functional activity of many drug-metabolizing enzyme pathways and possess the capacity of active efflux of marker substrates into bile canalicular compartments. The uptake of low-density lipoprotein (LDL) [41] and the uptake and secretion of indocyanine green (ICG) [41] are specific to hepatocytes and, thus, are used to determine hepatocyte-specific function. Glycogen accumulation, as examined by Periodic acid-Schiff staining, indicates the generation of mature hepatocytes [41].

In addition to the above-mentioned “hepatocyte-like” characteristics, both iPSCs and ESCs were differentiated into liver-like tissue with similar mitochondrial development as measured by oxygen concentration and pH in the culture medium, corresponding to the oxygen consumption rate and extracellular acidification rate, respectively [42]. The iPSCs had low oxygen consumption and possessed small, immature mitochondria located around the nucleus. With maturation to HLCs, mitochondria exhibited elongated morphology, swollen cristae, and dense matrices as well as cytoplasmic migration, increased mitochondrial DNA transcription- and replication-related gene expression, and increased oxygen consumption [43]. Although efficient hepatic differentiation from mouse iPSCs was observed, mouse iPSCs showed relatively lower hepatic induction efficiency compared with mouse ESCs [44]. Notably, HLCs persistently express alpha fetoprotein and lack key mature hepatocyte functions, as reflected by the drastically reduced activity of many detoxification enzymes [45,46,47]. These subtle but important differences between HLCs and adult hepatocytes have limited the use of stem cells as a renewable source of functional hepatocytes. The seven major metabolic pathways (oxidation, dehydrogenation, ketone formation, and potential methylation in phase I and glucuronidation and conjugations of glucose and glutathione in phase II) of the drug bufuralol found in iPSC-derived HLCs resembled those of freshly isolated primary human hepatocytes [48]. However, an enrichment analysis of apoptotic pathways found that iPSC-derived HLCs are more similar to cryopreserved human hepatocytes than the hepatic cell lines HepaRG and Huh7 [49]. During the hepatic differentiation process *in vitro*, these markers and metabolic markers may help scientists to control the differentiation process and obtain particular target cells for applications *in vitro* or *in vivo*.

## 5. *In Vitro* Differentiation of iPSCs to HLCs

**Figure 3:**
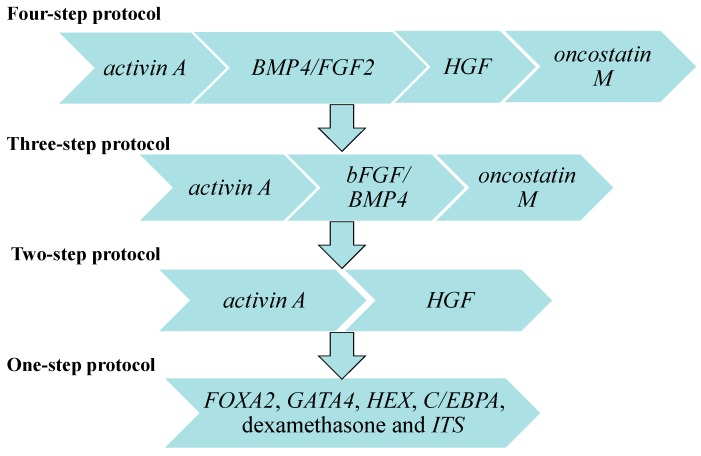
General protocols for *in vitro* hepatic differentiation of iPSCs.

By sequential stimulation with cytokines that are known to play a role in liver development, mouse iPSCs can be differentiated into two types of cells: one type with hepatoblast features and another type with hepatocyte characteristics [31]. Human liver development involves anterior endoderm differentiation, definitive endoderm formation, hepatoblast generation, and fetal or adult hepatocyte maturation [50]. Hepatic differentiation of iPSCs always occurs in a stepwise manner with high efficiency and reproducibility; however, simpler protocols of directed differentiation toward HLCs need to be developed for clinical usage [45,51,52,53] (Figure 3). Using a four-step differentiation protocol, human iPSCs were differentiated to definitive endoderm in response to *activin A* and exhibited hepatic specification in response to *BMP4/FGF2*, hepatoblast formation in response to *HGF*, and hepatocyte-like differentiation in response to *oncostatin M* [45]. By following a three-step differentiation protocol using *activin A*, *bFGF*, *BMP4*, and *oncostatin M*, the resulting iPSC-derived HLCs could represent a valuable hepatocyte source [51]. The hepatic differentiation efficiency of another two-step protocol with high doses of *activin A* and *HGF* was comparable to the four-step induction protocol [52]. Exposure to *FOXA2*, *GATA4*, *HEX*, *C/EBPA*, dexamethasone and *ITS* supplementation potentially serves as a single-step inducer for the differentiation of iPSCs into hepatic progenitor-like cells within eight days [53].

### 5.1. Differentiation to Immature HLCs

The differentiation propensity toward definitive endoderm was affected by donor origin but not by reprogramming methods or cell origin [54]. Successful promotion of iPSCs to a definitive endoderm fate at high efficiency and low cell density during induction is vital for the differentiation efficiency [55]. The utilization of an embryoid body step in cultures typically introduced an unpredictable degree of variability between hepatic differentiation attempts. To address this problem, monolayer culture, hepatocyte reporter construct, and specific cell surface marker selection [56] are useful protocols for distinguishing the purified populations for functional testing in a variety of hepatocyte transplantation assays. In the middle stage of iPSC hepatic differentiation, Ki67 immunostaining showed an abundance of proliferating hepatic progenitor cells [57]. Unfortunately, the cells in the middle stage were unable to be re-plated and recycled *in vitro* [37]; they could only be differentiated to terminal HLCs for numerous applications.

Currently, many cytokines and small molecules (signaling pathway regulators or epigenetic regulators) are able to influence the hepatic differentiation efficacy. PI3-kinase blocks the inductive effect of activin/nodal on definitive endoderm differentiation by controlling the activity of *WNT* signaling, and inhibition of this pathway significantly improves the differentiation of iPSCs to definitive endoderm [58]. The early differentiation to meso-endoderm and the subsequent distinction of mesenchyme and endoderm is driven primarily by the nodal, *BMP*, and activin signaling pathways [59]. A combination of human *activin A* with B27 minus insulin can efficiently differentiate human iPSCs into definitive endoderm at a rate of above 90% [60]. Under feeder- and serum-free conditions, overtreatment with CHIR99021 *c*onverted the cells into mesoderm cells rather than endoderm [55]. Human ESC-derived fibroblast-like cell-secreted factors act in concert with the central region of ESCs and are able to induce robust hepatic endoderm differentiation on iPSCs [61]. In addition, triiodothyronine induces Krüppel-like factor 9 in ESCs and iPSCs, and this effect persists during differentiation to definitive endoderm and hepatocytes [62]. With the help of poly(ε-caprolactone) nanofibrous scaffolds, definitive endoderm was able to be generated from iPSCs within a six-day induction period [63].

Initiated by hepatocyte differentiation-initiating medium, iPSCs were converted to hepatoblast-like cells, but a small number of immature hepatocytes was obtained [64]. To improve efficacy, a *HEX*-expressing adenovirus vector was used under serum/feeder cell-free chemically defined conditions, and iPSCs were efficiently induced to hepatoblasts [65]. The resulting cells expressed alpha fetoprotein at day nine and expressed albumin at day 12. Thus, developing new factors or clarifying the mechanism underlying the process is extremely helpful for the generation of functional HLCs.

### 5.2. Maturation of iPSC-Derived Hepatic Lineage Cells

As with immature HLC differentiation, the developmental pathways that control hepatocyte maturation from the fetal to the adult state are poorly understood; this issue has hampered the clinical usage of these cells. By culturing embryoid bodies in specific cytokine cocktails or on different extracellular matrices in an effort to mimic signals observed during embryonic liver development, the efficiency of iPSC-derived HLC generation can be improved [66].

The introduction of human *HNF6* markedly increased the expression of *CYP3A4* and other *CYP* genes in iPSC-derived HLCs as well as in HepG2 cells and hepatocytes, but the hepatocyte-specific gene expression levels in iPSC-derived HLCs were very low compared with those in HepG2 cells and hepatocytes [67]. *FOXA2* and *HNF1A* promoted efficient hepatic differentiation from human ESCs and iPSCs, and the expression profiles of hepatic marker genes were comparable to those of primary human hepatocytes [68]. Adenovirus vector-mediated over-expression of *HNF4A* in induced hepatoblasts led to the up-regulation of epithelial and mature hepatic markers and promoted hepatic maturation by activating mesenchymal-to-epithelial transition [69]. When knocking in a monomeric Kusabira-Orange (*mKO1*) cassette in the albumin gene for human ESCs and iPSCs using helper-dependent adenovirus, the hepatic marker genes, drug metabolism genes, and many aspects of liver function were highly enriched in the *ALB/mKO1* (Hi) population [70]. Efficiency can also be improved without exogenous factors. For example, with the addition of human serum from patients undergoing hepatectomy [71], differentiated cells expressed hepatic marker genes and drug-metabolizing enzymes and exhibited drug-metabolizing enzyme activities.

Shan *et al.* [72] developed a high-throughput screening platform for primary human hepatocytes to identify two different classes of small molecules that can be used to generate renewable sources of functional human hepatocytes. Valproic acid inhibited histone deacetylase activity during the differentiation of human iPSCs, and the HLCs expressed hepatic marker genes and drug-metabolizing enzymes and exhibited drug-metabolizing enzyme activities after 25 days of hepatic differentiation [73]. A similar phenomenon was observed in modified L-15 medium containing galactose, phenylalanine, and ornitine but deprived of glucose, tyrosine, arginine, and pyruvic acid [74]. Vitamin K2 and lithocholic acid activated pregnane X receptor, while albumin and apolipoprotein B100 were at levels equivalent to primary human hepatocytes [75]. Intriguingly, small molecules (CHIR99021, BIO, DMSO, dexamethasone, and dihexa) in a defined minimum medium without any of the exogenous growth factors necessary for hepatic specification promoted approximately 90% of iPSCs to HLCs [76,77]. Paracrine signals produced by the different subpopulations of liver-derived mesenchymal stem cells purified with immunoselection technologies have been identified and shown to induce the differentiation of human hepatic stem cells into fully mature and functional parenchymal cells [78]. Signaling pathways including *BMP4*, *FGF2*, and *FGF4* can induce differentiation to hepatoblasts; after liver bud formation and expansion, *HGF* and oncostatin signals stimulate the hepatoblasts to differentiate into hepatocytes [59]. In addition, *Wnt/β-catenin* signaling can induce the fate change of endodermal cells into a liver fate in a cell-autonomous manner [79].

### 5.3. Three-Dimensional (3D) Culture for Hepatic Differentiation

Under monolayer culture conditions, hepatocytes lose their liver-specific functions within a few days, leading to an extraordinary decrease in functional HLCs. iPSCs can be aggregated in suspension or on specialized plates, and this characteristic results in the formation of 3D structures that may serve to replicate some of the cell-cell and cell-matrix signals similar to hepatic development *in vivo* (Table 1) [80].

**Table 1:** iPSC hepatic differentiation under special 2D or 3D environments.

Species	Cell Type	Target Cell	2D/3D Culture	Efficiency	Reference
Human	iPSCs	Definitive endoderm	Poly(ε-caprolactone) nanofibrous scaffold	Reduced cell stress, rapid cell adaption, and high viability, growth, and differentiation	[63]
Human	iPSCs	Definitive endoderm	CHIR99021/Wnt3A ligand under feeder- and serum-free conditions	Easier handling and higher efficiency	[55]
Human	iPSCs	Hepatoblasts	Serum/feeder cell-free chemically defined conditions	Faster transition, more functional and higher efficiency	[65]
Mouse	iPSCs	HLCs	3D micro-cavitary hydrogel system	Nutrient exchange enhancement, greater living space, faster transition, and higher efficiency	[81]
Human	iPSCs	HLCs	Nanofiber scaffolds	More functional and higher efficiency	[82]
Human	iPSCs	HLCs	3D micropattern plate	More functional, higher efficiency, large number of HLCs for industrial and clinical applications	[83]
Human	iPSCs	HLCs	Scalable stirred-suspension bioreactor	Multiple features of primary hepatocytes and persistent function *in vivo*	[84]
Mouse	iPSCs	HLCs	Combination of a bioreactor module with a 0.2 μm pore membrane	Act as a promising option for bioartificial liver systems	[85]
Human	iPSCs	HLCs	3D collagen matrices compatible with high-throughput screening	Promote functional maturation and improve functional longevity to over 75 days	[86]
Human	iPSCs	HLCs	A micro-patterned co-culture platform	Promote functional maturation and improve functional longevity to over four weeks	[87]
Human	iPSC derived-hepatoblasts	HLCs	Combination of 3D cell aggregation and cAMP signaling	Comparable function to primary human hepatocytes, more simple and reproducible	[88]
Human	iPSC derived-hepatoblasts	HLCs	Human laminin 111-coated dish	Longevity of more than 3 months	[89]
Human	iPSCs	Liver bud	Coculture with endothelial cells and mesenchymal stem cells, and then mixed cells are plated onto a presolidified matrix	Fast formation of liver bud and act as a functional liver *in vivo*	[90]

Three-dimensional collagen matrices compatible with high-throughput screening [86], a micro-cavitary hydrogel platform system [81], and a micro-patterned co-culture platform [87] significantly increased the functional maturation of iPSC-derived HLCs toward an adult phenotype compared to conventional 2D systems. The combination of 3D cell aggregation and cAMP signaling enhanced the maturation of human iPSC-derived hepatoblasts to an HLC population that displays expression profiles and metabolic enzyme levels comparable to those of primary human hepatocytes [88]. Furthermore, by culturing on a human laminin 111-coated dish, human iPSC-derived hepatoblast-like cells were maintained for more than three months and had the ability to differentiate into both HLCs and cholangiocyte-like cells [89]. When iPSC-derived specified hepatic cells were dissociated and suspended with endothelial cells and mesenchymal stem cells and then plated onto a presolidified matrix [90], the immature endodermal cells self-organized into 3D liver buds by recapitulating the organogenetic interactions between endothelial and mesenchymal cells. The formation of functional vasculatures stimulated the maturation of iPSC liver buds into tissue resembling the adult liver [91].

## 6. Direct Reprogramming as an Alternative

Lineage reprogramming, which can be defined as the direct induction of functional cell types from one lineage to another lineage without passing through an intermediate pluripotent stage [92], avoids the potential problems associated with the time-consuming and labor-intensive generation of iPSC lines. Interestingly, the directly reprogrammed functional hepatocytes possess high proliferative potential, whereas such proliferative potential has not been reported for HLCs derived from iPSCs.

Zhu *et al.* did not generate iPSCs but instead cut short the reprogramming to pluripotency to generate an induced multipotent progenitor cell state. Subsequently, the HLCs could be efficiently differentiated [93]. By over-expressing the hepatic fate conversion factors *HNF1A*, *HNF4A*, and *HNF6* along with the maturation factors *ATF5*, *PROX1*, and *C/EBPA*, directly reprogrammed HLCs express a spectrum of phase I and II drug-metabolizing enzymes and phase III drug transporters. Importantly, the metabolic activities are comparable between reprogrammed HLCs and freshly isolated primary human hepatocytes [94]. In another study, over-expression of *GATA4*, *HNF1A*, and *FOXA3* along with *p19ARF* inactivation enabled mouse fibroblasts to become HLCs [95] that exhibited typical epithelial morphology, expressed hepatic genes, and acquired hepatocyte functions. A combination of *HNF4A* with *FOXA1*, *FOXA2*, or *FOXA3* was used to transfect and reprogram adult mouse fibroblasts to HLCs, and the efficiency was approximately one in 1000 of the initially transfected embryonic fibroblasts [96]. With repeated transfection of synthetic modified mRNAs (*HNF1A* plus any two of the factors *FOXA1*, *FOXA3*, or *HNF4A*), the directly reprogrammed cells also exhibited hepatic morphology and protein expression [97]. In addition to fibroblasts, spermatogonial stem cells (SSCs) can trans-differentiate to hepatic stem-like cells that are capable of differentiating into cells with the morphological, phenotypic, and functional characteristics of mature hepatocytes via activation of the *ERK1/2* and *SMAD2/3* signaling pathways and inactivation of *cyclin A*, *cyclin B*, and *cyclin E* [98]. Considering the omission of a pluripotent state and hepatic function comparable to primary hepatocytes, direct reprogramming to HLCs may act as a promising route to produce a large number of HLCs without the laborious generation of iPSCs for clinical usage.

## 7. *In Vivo* Hepatic Differentiation of iPSCs

Transplanted iPSCs engrafted, integrated, and proliferated in the liver and the surviving stem cells secreted albumin, indicating that iPSCs can differentiate to HLCs and function *in vivo* [48]. Preliminary transplantation experiments not only confirmed engraftment but also showed teratoma formation, which needs to be excluded using more stringent purification strategies [99]. Intravenous transplantation of iPSCs effectively reduced the hepatic necrotic area, improved liver function and motor activity, and rescued lethal acute liver failure [100]. Human iPSCs of distinct origins, regardless of their parental epigenetic memory, can efficiently differentiate along the hepatic lineage to regenerate mouse liver [101,102]. When iPSC-derived HLCs at different stages were transplanted, 15- and 20-day hepatic differentiated cells engrafted into the livers and further acquired hepatocyte morphology. In contrast, five- and 10-day differentiated cells were able to engraft but did not generate HLCs *in vivo* [103]. To enhance the repopulation efficacy, the apoptosis of human iPSC-derived HLCs during the transplantation process was inhibited by transducing with an adenovirus vector expressing *FNK* [104]. To clarify the potential therapeutic effects *in vivo*, scientists set out to compare the resulting functional HLCs from hepatic differentiation *in vivo* and *in vitro*. Transplantation of iPSCs performed better than iPSC-derived HLCs in reducing ALT and AST levels in the serum of patients and the liver necrosis areas [105], and the hepatoprotective effect appeared to originate from the higher antioxidant activity [106]. Both iPSCs and iPSC-derived HLCs potentially suppressed *ROS* production and activated antioxidant enzymes in injured livers to repair the injured liver [107].

In a 3D environment, iPSC-derived HLCs can efficiently rescue impaired liver function. When suspended with endothelial cells and mesenchymal stem cells and plated onto a pre-solidified matrix, iPSC-derived HLCs formed a 3D spherical tissue mass termed a liver bud and performed multiple hepatic functions in a chronological manner *in vivo* after transplantation [90]. Furthermore, mesenteric transplantation of liver buds rescued the drug-induced lethal liver failure model and performed liver-specific functions such as protein production and human-specific drug metabolism without recipient liver replacement [91]. With the delivery of miR122 and carboxymethyl-hexanoyl chitosan, the period of iPSC hepatic differentiation was shortened, and the resulting HLCs exhibited promising hepatoprotective efficacy *in vivo* [108].

Since various laboratories began to directly reprogram somatic cells into HLCs, they also detected the directly reprogrammed HLCs *in vivo* to simultaneously evaluate the application prospects. After transplantation into an immune-deficient mouse model of human liver failure, the directly reprogrammed HLCs proliferated extensively and acquired levels of hepatocyte function similar to those of primary hepatocytes. Unlike the differentiated HLCs, these reprogrammed HLCs did not form tumors most likely because they never entered a pluripotent state [93]. In particular, the directly reprogrammed HLCs were able to repopulate the livers of fumarylacetoacetate hydrolase-deficient mice and were sufficient to restore liver function and rescue 40% of recipient mice from death [95,96].

## 8. The Potential Applications of iPSC-Derived HLCs

Transplantation of liver lineage cells or tissue-engineered liver tissue analogs [107] and extracorporeal bioartificial liver devices [109] are two promising applications for HLC therapies. Nevertheless, these two applications are inhibited by oncogenesis and by cell scarcity. Evidence has shown that iPSCs frequently acquire aneuploidies and recurrent abnormalities [110]. These cells may retain the inactive X chromosome from the somatic cell source [111] or reactivate the inactive X and display two active X chromosomes [112]. Long-term culture has also been associated with malignant embryonic carcinoma cells [113]. Abnormal epigenetic modifications can ultimately affect the pluripotency of iPSCs; the disrupted methylation could not be recovered by optimizing culture conditions or by subcloning iPSCs. In addition, reactivation of *C-MYC* might result in tumor formation, but the absence of *C-MYC* drastically reduced the reprogramming efficiency and resulted in subtle abnormal epigenetic modifications [114]. Although the genomic integrity of iPSCs has limited their clinical applications, numerous reprogramming strategies were developed to minimize the disturbance of the iPSC genome. While aneuploidy is commonly associated with cell transformation, iPSCs that harbor abnormal chromosomal content retain the capacity to generate HLCs with high efficiency [115].

Currently, HLCs are extensively used as platforms for drug discovery [116] or for the determination of pharmaceutical-induced hepatotoxicity [117], as models of liver diseases [118], and as individual therapies in patients [119]. To determine individual drug effects on disease-specific iPSC-derived functional cell types, the disease phenotypes need to be recapitulated consistently and uniformly. Disease modeling conditions must be further developed and differentiation conditions must be further optimized for more accurate disease modeling and drug screening. iPSCs were generated from a cohort carrying mutations (PiZZ) in the gene responsible for α-1 antitrypsin (*AAT*) deficiency. The expression of 135 genes distinguished PiZZ iPSC-derived HLCs, which displayed intracellular accumulation of mutant *AAT* protein, resulting in increased autophagic flux but adverse responses to known hepatotoxic drugs [120]. Through a blind large-scale drug screening, five clinical drugs were identified that reduced *AAT* accumulation in patient iPSC-derived HLCs. In addition, using the recently developed transcription activator-like effector nuclease technology, Choi *et al.* achieved a high gene-targeting efficiency in *AAT*-deficient patient iPSCs, with 25%–33% of the clones demonstrating simultaneous targeting at both diseased alleles [121]. HLCs obtained from patient-derived iPSCs could also be used as a platform for individualize therapies without immune suppression. Meanwhile, many congenital liver diseases benefit from these reprogrammed cell-based therapies. HLCs were found to recapitulate key pathological features of the diseases affecting the patients from which they were derived [119]. Combined with directed cell differentiation strategies, Wilson’s disease-derived iPSCs were successfully differentiated into HLCs, neural stem cells, and neurons, accompanied by the expression of the mutant *ATP7B* gene in all differentiated cells [122]. When combined with the capacity to engineer genetic changes in established iPSCs [123], patient-specific iPSCs and HLCs can be utilized to study genetic variants identified in Genome-wide association studies (GWAS) studies as well as a host of other monogenic alterations to assay their impact on hepatocyte differentiation, phenotype, and function [124], thus facilitating the *in vitro* tmodeling of rare diseases. In addition, the patient-derived cells will also facilitate the direct investigation of hepatitis B and C viruses in human hepatocytes and the determination of the genetic influence on the susceptibility of hepatocytes to these viruses [125]. After improving the differentiation efficacy and eliminating genomic disturbance, these somatic cell-derived HLCs may one day enable personalized medicine.

## 9. Conclusions

In summary, recent technical advances in reprogramming somatic cells to iPSCs with different reprogramming systems and alternative combinations of transcription factors will lead to numerous pluripotent stem cells without gene modifications. In addition, feeder-free culture systems and 3D culture systems play extremely important roles in the generation of clinical-grade pluripotent cells. However, the incomplete hepatic differentiation state of iPSCs likely results from our poor understanding of the mechanisms underlying the developmental shift from fetal to adult liver. Thus, after clarifying the characteristics of different hepatic differentiation stages, the handling of this process will be easier and more efficient. The differentiation protocols combined with the development of complex culture systems will significantly improve the quality and spectrum of iPSC-derived hepatocyte-like cell types. In contrast, the direct reprogramming of HLCs that bypass the pluripotent state could occur efficiently *in vitro* and *in vivo*. As these novel technologies become rapidly available in the reprogramming or differentiation field, they may enable studies at the deepest molecular level, linking genetics, epigenetics, and phenotypes in the context of complex liver disorders. Although iPSCs are accompanied by abnormal chromosomes, these pluripotent cells remain able to differentiate to mature HLCs. It is likely that the generation of safe and effective HLCs for cell therapy as well as disease modeling and drug screening will be possible in the near future.

## Abbreviations

ESCs: embryonic stem cells
iPSCs: induced pluripotent stem cells
HLCs: hepatocyte-like cells
LDL: low-density lipoprotein
ICG: indocyanine green
mKO1: monomeric Kusabira-Orange
3D: three-dimensional
SSCs: spermatogonial stem cells
AAT: alpha-1 antitrypsin
